# A Nationwide Antenatal Human T-Cell Leukemia Virus Type-1 Antibody Screening in Japan

**DOI:** 10.3389/fmicb.2020.00595

**Published:** 2020-04-09

**Authors:** Kazuo Itabashi, Tokuo Miyazawa, Akihiko Sekizawa, Akifumi Tokita, Shigeru Saito, Hiroyuki Moriuchi, Yasuhito Nerome, Kaoru Uchimaru, Toshiki Watanabe

**Affiliations:** ^1^Showa University Hospital, Tokyo, Japan; ^2^Department of Pediatrics, Showa University School of Medicine, Tokyo, Japan; ^3^Japan Association of Obstetricians and Gynecologists, Tokyo, Japan; ^4^Japanese Pediatric Association, Tokyo, Japan; ^5^The University of Toyama, Toyama, Japan; ^6^Department of Pediatrics, Nagasaki University Graduate School of Biomedical Sciences, Nagasaki, Japan; ^7^Faculty of Medicine, School of Health Sciences, Kagoshima University, Kagoshima, Japan; ^8^Graduate School of Frontier Sciences, The University of Tokyo, Tokyo, Japan; ^9^Future Center Initiative, and Research Hospital of the Institute of Medical Science, The University of Tokyo, Tokyo, Japan

**Keywords:** human T-cell leukemia virus type-1, nationwide antenatal screening, confirmatory test, mother-to-child transmission, infection, prevention

## Abstract

Japan has been running a nationwide antenatal human T-cell leukemia virus type-1 (HTLV-1) antibody screening program since 2010 for the prevention of HTLV-1 mother-to-child transmission. As part of the program, pregnant women are invited to take an HTLV-1 antibody screening test, usually within the first 30 weeks of gestation, during regular pregnancy checkups. Pregnant women tested positive on the antibody screening test undergo a confirmatory test, either western blotting or line immunoassay. In indeterminate case, polymerase chain reaction (PCR) is used as a final test to diagnose infection. Pregnant women tested positive on a confirmatory or PCR test are identified as HTLV-1 carriers. As breastfeeding is a predominant route of postnatal HTLV-1 mother-to-child transmission, exclusive formula feeding is widely used as a postnatal preventive measure. Although there is insufficient evidence that short-term breastfeeding during ≤3 months does not increase the risk of mother-to-child transmission compared to exclusive formula feeding, this feeding method is considered if the mother is eager to breastfeed her child. However, it is important that mothers and family members fully understand that there is an increase in the risk of mother-to-child transmission when breastfeeding would be prolonged. As there are only a few clinical studies on the protective effect of frozen-thawed breastmilk feeding on mother-to-child transmission of HTLV-1, there is little evidence to recommend this feeding method. Further study on the protective effects of these feeding methods are needed. It is assumed that the risk of anxiety or depression may increase in the mothers who selected exclusive formula feeding or short-term breastfeeding. Thus, an adequate support and counseling for these mothers should be provided. In addition to raising public awareness of HTLV-1 infection, epidemiological data from the nationwide program needs to be collected and analyzed. In most cases, infected children are asymptomatic, and it is necessary to clarify how these children should be followed medically.

## Introduction

While the majority of HTLV-1-infected individuals remain asymptomatic, the two well-recognized disease associations ATL and HAM/TSP are caused by the virus. HTLV-1 carriers are estimated to have a lifetime risk of 2–7% for the development of ATL (Iwanaga et al., 2012) and 0.25–3.8% for HAM/TSP (Yamano and Sato, 2012). Both these diseases exhibit serious clinical manifestations, and the associated prognosis remains poor despite therapeutic efforts (Katsuya et al., 2015; Willems et al., 2017). Numerous studies have demonstrated that MTCT through breastfeeding is the predominant route of HTLV-1 infection (Hino et al., 1987; Murphy et al., 1989; Hino, 2011), while HAM/TSP develops in both populations infected via vertical and horizontal routes (Bartholomew et al., 1998). Thus, antenatal HTLV-1 screening program is expected to play an important role, especially in reducing the number of ATL patients.

A first step in taking measures to prevent HTLV-1 MTCT is to determine whether the mother is infected. To date, there are no effective measures to prevent antenatal infection, but avoiding or restricting breastfeeding is expected to reduce the number of postnatal infections via MTCT. In turn, the prevalence of HTLV-1-associated diseases could be reduced, and the rising trend in the number of people with horizontal infection could be curbed to some extent. Non-endemic and endemic countries may have different views on the need to introduce a nationwide screening program, but in countries or areas where HTLV-1 is endemic, antenatal screening is likely to contribute to a reduction in the burden of associated diseases (Ribeiro et al., 2012; Rosadas et al., 2018).

In 2010, the Ministry of Health, Labor, and Welfare in Japan decided to conduct a nationwide HTLV-1 antibody screening program for all pregnant women (Nishijima et al., 2019). Japan is the first country in the world to conduct such a nationwide screening program. There are several factors to this— (1) Japan is the only developed country with >1 million HTLV-1 carriers (Satake et al., 2012); (2) HTLV-1 carriers are spreading throughout Japan due to internal population migration (Satake et al., 2012); (3) >4,000 adolescents and adults (77% female) are newly diagnosed annually (Satake et al., 2016); and (4) to date, no effective vaccines or antiviral regimens have been developed yet (Willems et al., 2017).

The United Kingdom National Screening Committee had considered antenatal HTLV-1 screening program three times, but the committee did not recommend introducing a screening program in the United Kingdom because of the low prevalence of HTLV-1 infection and the low risk for infected infants to develop a serious illness. The Committee maintained its conclusions after updating and reviewing the evidence in 2017 (UK National Screening Committee, 2017). However, Malik and Taylor (2019) analyzed the cost-effectiveness of a United Kingdom screening program using a highly conservative model of transmission and disease attribution. This analysis suggested that an antenatal screening program to identify HTLV-1 carriers and reduce transmission was potentially cost-effective in the United Kingdom.

In this review, we would like to introduce the nationwide antenatal screening program in Japan and discuss the associated issues.

## Antenatal Mother Screening for HTLV-1 Antibody

### Algorithm for Virus Carrier Screening

**FIGURE 1:**
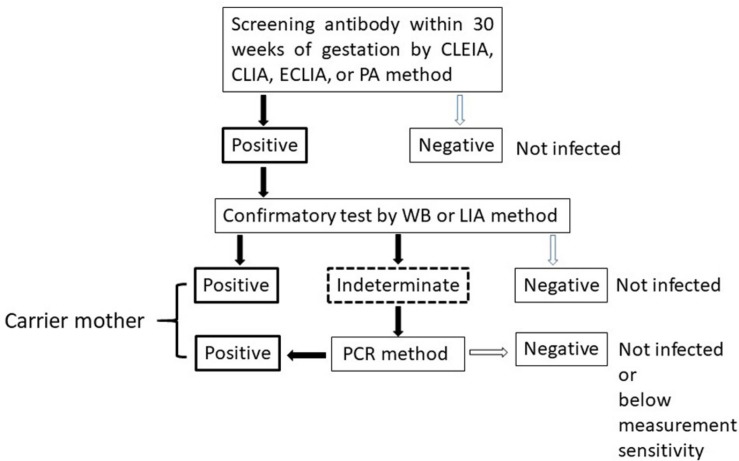
Algorithm to determine HTLV-1 virus carriers among pregnant women. CLEIA, chemiluminescent enzyme immunoassay; CLIA, chemiluminescent immunoassay; ECLIA, electro chemiluminescent immunoassay; PA, particle agglutination; WB, Western Blot; LIA, line immunoassay; PCR, polyclonal chain reaction.

The algorithm for HTLV-1 carrier screening during pregnancy in Japan is shown in Figure 1. HTLV-1 antibody screening is usually performed within the first 30 weeks of gestation to secure enough time for a carrier to gain access to the detailed information from healthcare providers and to select a suitable feeding method before labor. Confirmatory tests are performed for pregnant women with positive screening results. In indeterminate cases, PCR is used as a definite test to diagnose infection. Pregnant women who have either a positive confirmatory test or PCR-positive results are identified as HTLV-1 carriers.

### Assays for HTLV-1 Antibody Screening

In Japan, laboratory screening for HTLV-1 infection has been routine practice for blood donors since 1986 (Inaba et al., 1989). Furthermore, following several cases of HAM/TSP and ATL in donors and recipients after organ transplantation, HTLV-1 screening has been proposed for both transplant donors and recipients (Gallo et al., 2016; Kawano et al., 2018; Moreno-Ajona et al., 2018).

Several assays for HTLV-1 antibody screening are available, including PA (Fujino et al., 1991), CLEIA (Morota et al., 2009), CLIA (Qiu et al., 2008), and ECLIA (Laperche et al., 2017). These assays are available in Japan because they are capable of processing large numbers of samples in a relatively short time. A multicenter performance evaluation study in Europe and Japan was carried out with the new ECLIA for HTLV-I/II antibody detection (Laperche et al., 2017). This study demonstrated a specificity of 99.83% and sensitivity of 100% in routine diagnostic samples, regardless of the geographic origin of the samples, the virus type, or the location of the testing laboratory. This assay has the sensitivity and specificity to support its use as a routine screening assay for detecting HTLV infection. The development of screening assays with high sensitivity and specificity has contributed to HTLV-1 detection.

However, antibody screening tests use different antigens and have different measurement principles, and the test results often do not match between them due to the methods used. In addition, these tests have a high false-positive rate, especially in non-endemic areas. For this reason, a confirmatory test must be performed following a positive screening test.

### Confirmatory Test

According to data collected retrospectively by the Japan Association of Obstetricians and Gynecologists, the prevalence rate of pregnant women tested positive on a PA or CLEIA screening test was 0.32% (2,259/707,711) in 2011. Among 2,259 pregnant women who screened positive, 1,894 women (83.8%) underwent a WB test as a confirmatory test. Thus, the screening program was still in its early days, and confirmatory tests were not performed on all cases.

The number of WB positive, indeterminate, negative, and missing cases was 942 (49.7%), 212 (11.1%), 660 (34.8%), and 80 (4.2%), respectively. The rate of false-positive results was 14.0% (88/629) in Kyushu and Okinawa prefecture, which are endemic areas in Japan, whereas it was 45.2% (572/1,265) in other areas (Suzuki et al., 2014). The results show that the positive predictive value of any screening assay is low in non-endemic areas and generates a substantial number of false-positive results, highlighting the need for a confirmatory test (Morrison et al., 2015).

Western Bolt is the approach that has been the most frequently used for the confirmatory test. WB measures the serological reaction to both Gag core proteins (p19, p24, and p53) and the Env protein gp46 (WHO News and Activities, 1991). Unfortunately, WB exhibits a high proportion of indeterminate results (Garin et al., 1994; Filippone et al., 2012; Suzuki et al., 2014). Kuramitsu et al. (2017) explored the reasons why WB methods show a high proportion of indeterminate results. They revealed that the maximum proviral load (PVL) in WB-indeterminate samples from pregnant women was 1 copy/100 peripheral blood mononuclear cells (PBMCs), and the median (0.01 copy/100 PBMCs) was approximately 100-fold lower than that of WB-positive samples, as determined by a PCR assay (Kuramitsu et al., 2017). They also reported that the proportion of HTLV indeterminates with detectable provirus was 16.5% (32/194) among pregnant women. Such carrier status may have a very low risk of developing ATL because the PVL is significantly lower than that necessary for the development of the disease (>4 copies/100 PBMCs) (Iwanaga et al., 2010). The authors also observed mutations in the provirus which would interfere with host recognition of HTLV-1 antigens. Thus, they suggested that WB-indeterminate carriers have a low production of viral antigens due to these mechanisms.

Recently, the LIA has been implemented in Japan in replacement of WB. LIA was developed for the serological confirmation and discrimination of HTLV-1 and -2 infection (Zrein et al., 1998). This assay performs well in confirming HTLV-1 seropositivity by exhibiting a low incidence of indeterminate results. Further, the results are in good agreement with PCR results (Sabino et al., 1999; Umeki et al., 2017). It was reported that the number of indeterminate results was reduced by up to 90% when LIA was introduced to replace the WB confirmatory test (Thorstensson et al., 2002). Thus, LIA may be expected to decrease the costs of diagnosis.

However, PCR should be conducted for determining PVL in those cases where the confirmatory tests show indeterminate results. Nowadays, both LIA and qualitative PCR test are covered by the Universal Health Insurance system in Japan as part of the antenatal HTLV-1 screening program. If the PCR qualitative test is negative, it means that there is no infection or that the PVL is below the sensitivity of measurement (<4 copies/10^5^PBMCs).

## HTLV-1 Prevalence Among Pregnant Women

The nationwide prevalence of HTLV-1 infection is generally estimated using blood donor data. Health studies on blood donors can be affected by a selection bias due to the healthy donor effect, in which donors are generally healthier than the general population (Atsma et al., 2011). Thus, the estimated number of HTLV-1 infected people might be underestimated. On the other hand, studies of pregnant women may have a bias in the opposite direction compared to studies of blood donors because of sexual intercourse with infected partner. The seroprevalence of HTLV-1 and HTLV-2 in Western Europe is 6-fold higher among pregnant women (4.4 per 10,000) than that among blood donors (Taylor et al., 2005). Although the two populations were surveyed at different times in Japan, the prevalence rate among women in a 2005–2006 study of blood donors was 6.88 per 10,000 (Satake et al., 2012) compared to 16 per 10,000 for pregnant women in 2011 (Suzuki et al., 2014). More detailed information on prevalence in several countries of HTLV-1 infection in pregnant women is summarized in the review written by Rosadas and Taylor (2019). However, many of these reports are limited to endemic countries and areas.

## Feeding Methods as a Postnatal Preventive Measure

To date, there have been no randomized controlled trials investigating HTLV-1 MTCT rates by feeding method. All previous reports are observational studies, and the number of cases per study is often small.

### Exclusive Formula Feeding

Since the main infection route of HTLV-1 MTCT is breastfeeding, it is reasonable to recommend avoiding breastfeeding. The ATL Prevention Program in Nagasaki revealed a marked reduction of HTLV-1 MTCT by ExFF from 20.3 to 2.5% (Hino, 2011). Nowadays, ExFF has been considered as the most reliable method for MTCT prevention (Ribeiro et al., 2012; Rosadas and Taylor, 2019).

### Short-Term Breastfeeding

In Japan, the debate on the use of STBF on MTCT prevention has continued since the 1990s. It has been pointed out that the risk of MTCT is lower in STBF than in longer term breastfeeding (Takahashi et al., 1991; Maehama et al., 1992; Oki et al., 1992; Takezaki et al., 1997; Wiktor et al., 1997; Ureta-Vidal et al., 1999; Takezaki, 2009; Hino, 2011). One of the reasons may be that antibodies against HTLV-1 are transferred from the carrier mother *in utero* and block MTCT for several months after birth (Takahashi et al., 1991). However, the presence of antibodies decreases over the first few postnatal months of life, so HTLV-1 infection may occur when breastfeeding is prolonged. Another reason may be that the cumulative number of infected cells entering the gastrointestinal tract is limited due to short-term breastfeeding. It has been proposed that an infant can ingest a total of 10^8^ HTLV-1 infected cells before weaning (Yamanouchi et al., 1985). In contrast, substances contained in breastmilk such as tumor growth factor-β and lactoferrin, which are rich in colostrum (Albenzio et al., 2016; Morita et al., 2018), and prostagrandin E_2_ have a promoting effect on HTLV-I replication (Moriuchi and Moriuchi, 2001, 2002; Moriuchi et al., 2001). If STBF could be effective to prevent postnatal MTCT, the antibodies transferred to the fetus *in utero* may overcome the enhanced viral replication during the first few months of life.

The ATL Prevention Program in Nagasaki from 1987 to 2004 showed an 7.4% (15/202) incidence of MTCT in children that were breastfed for <6 months. This was significantly higher than the rate of MTCT on ExFF (2.5%, 29/1,152; *P* < 0.001), but significantly lower than that on longer term (≥6 months) breastfeeding (20.3%, 74/365; *P* < 0.001) (Hino, 2011). Therefore, the ATL Prevention Program in Nagasaki has recommended ExFF for carrier mothers. According to previous studies, the rates of MTCT in children fed by short-term breastmilk during less than 7 months ranged from 3.4 to 9.8%, while ranged from 0 to 6.0% in children fed by exclusive formula. On the other hand, the MTCT rate tends to increase from 11.3 to 25% in longer-term breastfeeding (Table 1 and Supplementary Table S1; Takahashi et al., 1991; Nakayama et al., 1992; Oki et al., 1992; Takezaki et al., 1997; Ureta-Vidal et al., 1999; Hino, 2011).

**TABLE 1:** Comparison of mother-to-child transmission rates by exclusive formula feeding, short-term breastfeeding (<7 months) and longer-term breastfeeding.

Author, year	Study area	Study period	Exclusive formula feeding	Short-term breast feeding		Longer-term breastfeeding		Study design
						
			Seroconversion n/N (%)	Inclusion	Seroconversion n/N (%)	Inclusion	Seroconversion n/N (%)	
Takahashi et al., 1991	Kagoshima, Japan.(13 hospitals)	1985–1990	0/0(0%)	≤6 months	3/67 (4.5%)	>6 months	19/136(14.0%)	Retrospective
Takahashi et al., 1991	Kagoshima, Japan.(13 hospitals)	1986–1990	9/151(6.0%)	≤6 months	1/23 (4.3%)	>6 months	1/3(33.3%)	Prospective
Nakayama et al., 1992	Kagoshima, Japan.(single center survey)	1986–1990	1/53(1.9%)	≤6 months	4/41 (9.8%)	7–12 months	7/50(14.0%)	Retrospective
Oki et al., 1992	Kagoshima and Miyazaki, Japan	1986–1990	0/7(0%)	<7 months	3/67 (4.5%)	≥7 months	19/136(14.0%)	Retrospective
Oki et al., 1992	Kagishima and Miyazaki, Japan	1986–1991	10/177(5.6%)	<7 months	1/26 (3.8%)	≥7 months	1/4(25.0%)	Prospectiver
Takezaki et al., 1997	Tsushima and Kamigoto, Nagasaki, Japan	1985–1991	4/162(2.5%)	≤6 months	2/51 (3.9%)	>6 months	13/64(20.3%)	Retrospective
Ureta-Vidal et al., 1999	French Guyana	1989-NA	0/23(0%)	≤6 months	2/32 (3.4%)	>6 months	17/151(11.3%)	Retrospective
Hino, 2011	Nagasaki, Japan	1987–2004	29/1,152(2.5%)	<6 months	15/202 (7.4%)	≥6 months	74/365(20.3%)	Retrospective

*NA: not applicable.*

Several studies have shown that the rates of MTCT with ≤3 months of STBF ranged from 0 to 8.5% (Table 2 and Supplementary Table S2; Hirata et al., 1992; Ureta-Vidal et al., 1999; Kashiwagi et al., 2004; Takezaki, 2009; Moriuchi et al., 2017), while ranged from 0 to 12.8% in children fed by exclusive formula. On the other hand, the MTCT rate ranged from tends to increase from 5 to 28.6% in longer-term breastfeeding. Hirata et al. showed that the prevalence of HTLV-l antibody among children breastfed for over 3 months was significantly higher (16/28, 27.6%) than that of those breastfed for under 3 months (2/39, 5.1%; *P* = 0.012; Hirata et al., 1992). Based on these reports, some healthcare providers in Japan considered that STBF for up to 3 months is unlikely to increase the risk of MTCT and have therefore recommended STBF for ≤3 months if the carrier mother eager to breastfeed her infant. However, there is insufficient evidence for this speculation because almost these reports had the small sample size of studied children and the risk of bias due to selections of participants, confounding variables, and incomplete outcome data. And, it is unclear whether the risk of MTCT is clearly increased between 4 and 6 months. Further study is needed on the protective effects of STBF on MTCT.

**TABLE 2:** Comparison of mother-to-child transmission rates by exclusive formula feeding, short-term breastfeeding (≤3 months) and longer-term breastfeeding.

Author, year	Study area	Study period	Exclusive formula feeding	Short-term breastfeeding (≤3 months)		Longer-term breastfeeding		Study Design
						
			Seroconversion n/N (%)	Inclusion	Seroconversion n/N (%)	Inclusion	Seroconversion n/N (%)	
Ureta-Vidal et al., 1999	French Guyana	1989-NA	0/23(0%)	≤3 months	1/12 (8.3%)	>3 months	18/168(10.7%)	Retrospective
Hirata et al., 1992	Ishigaki island, Okinawa, Japan	1989–1991	10/78(12.8%)	≤3 months	2/39 (5.1%)	>3 months	16/58(21.6%)	Retrospective
Kashiwagi et al., 2004	Okinawa, Japan	1995–1999	1/31(3.2%)	≤3 months	1/25 (4.0%)	>3 months	1/20(5%)	Prospective
Takezaki, 2009	Kagoshima, Japan	1986–2006	16/331(4.8%)	≤3 months	2/126 (1.6%)	>3 months	9/46(19.6%)	Retrospective
Moriuchi et al., 2017	Nagasaki, Japan	2011–2017	4/91(4.4%)	≤3 months	3/35 (8.5%)	>3 months	6/21(28.6%)	Retrospective

*NA: not applicable.*

As children with longer duration of breastfeeding have higher rates of MTCT (Rosadas et al., 2018), it should be noted that MTCT rate in the longer-term breastfeeding group depends on the distribution of breastfeeding duration in the included subjects.

### Frozen-Thawed Breastmilk Feeding

There are very few studies evaluating the incidence of MTCT when using FTBMF. Ando et al. (1989) observed that infected cells in breast milk were effectively destroyed *in vitro* due to the process of freezing and thawing. The rate of MTCT on FTBMF in previous studies ranged from 0 to 7.1% (Ando et al., 1989, 2004; Maehama et al., 1992; Ekuni, 1997). Only two studies compare the effect of ExFF with that of FTBMF on the prevention of MTCT (Table 3 and Supplementary Table S3; Maehama et al., 1992; Ekuni, 1997). It however remains unclear whether FTBMF is effective in preventing MTCT because of the limited number of studies and participants.

**TABLE 3:** Comparison of mother-to-child transmission rates by exclusive formula feeding, frozen-thawed breastmilk feeding and breastfeeding.

Author, year	Study area	Study.period	Exclusive formula feeding	Frozen-thawed breast milk feeding		Breastfeeding		Study Design
						
			Seroconversion n/N (%)	Inclusion	Seroconversion n/N (%)	Inclusion	Seroconversion n/N (%)e	
Maehama et al., 1992	Okinawa, Japan	1986–1989	0/46(0%)	12 h freezing in a home freezer	2/26 (7.7%)	0–4 months	4(4.2%)	Retrospective
						5–8 months	2(7.4%)	
						9–12 months	1(4.2%)	
						≥13 months	3(16.7%)	
Ekuni, 1997	Okinawa, Japan	1983–1984	5/108(4.6%)	12 hfreezing at −20°C	0/33 (0%)	NA	13(41.9%)	Retrospective

*NA: not applicable.*

### Other Feeding Methods

Regardless of its duration, breastfeeding may also be combined with the use of infant formula. In recent studies of MTCT of HIV, MTCT rates with ordinary breastfeeding and ExFF were 2.70 and 3.77%, respectively, compared to 20.0% with mixed feeding (Njom Nlend et al., 2018). It is speculated that mixed feeding may cause gastrointestinal mucosal injury or dysbiosis, which may involve changes in intestinal permeability (O’Sullivan et al., 2015). However, to date, there is no evidence to inform mixed feeding recommendations to HTLV-1 carrier women, and further studies on the impact of mixed feeding on HTLV-1 MTCT are warranted.

## Strategy for Prevention Against HTLV-1 MTCT

**FIGURE 2:**
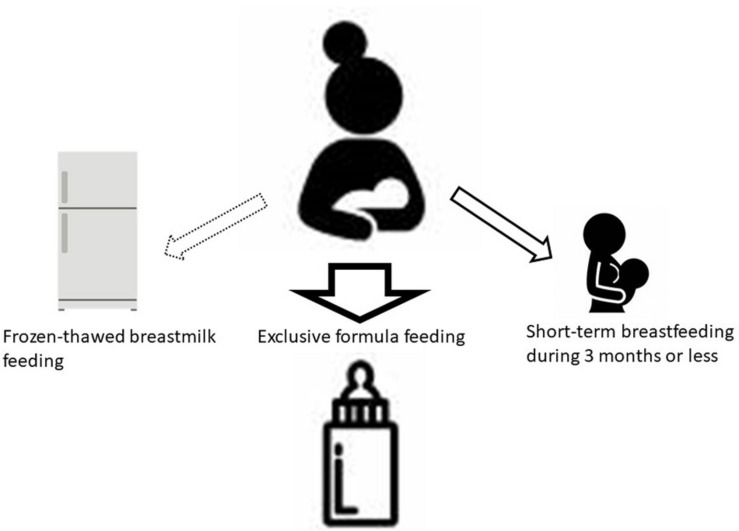
Selection of feeding methods. Due to the well-established evidence, ExFF should be the first choice for postnatal prevention of MTCT. If a carrier mother strongly desires to breastfeed, STBF during 3 months or less would be better to be selected. Health care providers should support her to avoid longer-term breastfeeding because prolonged periods may increase the risk of MTCT. There are few studies on the effects of FTBMF compared to ExFF. For preterm low birth weight infants, FTBMF using own mother’s milk during tube feeding would be better to be selected in consideration of reducing the risk of severe infections and necrotizing enterocolitis. Currently, the breast milk banking system is not available in Japan, but banked human milk is the best choice when it becomes available. ExFF, exclusive formula feeding; STBF, short-term breastfeeding; MTCT, mother-to-child transmission; FTBMF, frozen-thawed breastmilk feeding (Itabashi, 2016).

Even after the national antenatal HTLV-1 antibody screening test began in 2010, healthcare providers in each prefecture were instructing carrier mothers to choose among ExFF, STBF, and FTBMF for the next 5 years. However, within the same endemic area in Kyushu, Japan, STBF during ≤3 months or ExFF has been recommended in Kagoshima Prefecture (Nerome et al., 2014), while ExFF has been recommended mainly in Nagasaki Prefecture (Hino et al., 1994; Moriuchi et al., 2013). The selection of feeding methods by the carrier pregnant women is most likely influenced by the opinions of the healthcare providers. Therefore, we designated the strategies for prevention of HTLV-1 MTCT (Figure 2) in the manual of nationwide antenatal HTLV-1 screening program with the support of the Ministry of Health, Labor, and Welfare in 2016 (Itabashi, 2016). In this strategy, ExFF should be prioritized with the view to prevent postnatal MTCT. The STBF during ≤3 months rather than <7 months would be better to be selected if the mother is eager to breastfeed. However, it is important that mothers and family members fully understand an increase in MTCT risk with increased duration of breastfeeding and an insufficient evidence of this feeding method. Thus, a support system to help mothers to refrain from breastfeeding after 3 months of life may be necessary. There are few studies on the risk of MTCT by FTBMF compared to ExFF, and there is little evidence to recommend this feeding method. Considering the efforts needed by mothers in preparing frozen-thawed breastmilk represents every day, it may be better to use it only for preterm infants staying in newborn intensive care units. To date, there are no reports on the risk of MTCT by mixed feeding, which should be considered in the future.

## Issues Needed to Maximize the Effects of the Nationwide Screening Program

In Japan, HTLV-1 antibody testing is mandatory along with testing for other infectious diseases during health checkups for pregnant women. Although there is no specific data on the implementation rate, it is likely that most pregnant women have been tested for HTLV-1 antibody screening, except for those who have never undergone a prenatal checkup. There are several issues not only selection of feeding methods to prevent HTLV-1 MTCT but also the others to succeed the nationwide antenatal screening program and need to be solved in the future (Table 4). We have already discussed the selection of feeding methods, so we will discuss other issues here.

**TABLE 4:** Issues needed to maximize the effects of the nationwide screening program.

Issues	Countermeasures
Selection of feeding methods	Establishment of evidence on the prevention of MTCT by STBF and FTBMF
Evaluation of effect of mother screening on MTCT prevention	To increase the rate of antibody testing after 3 years of age
Public awareness	Necessary for patient groups, scientists, clinicians, and policy makers to work together to raise public awareness about HTLV-1 infection.
Support for carrier mothers	Establishment of adequate support system for carrier mothers in each prefecture
Elimination or reduction of the benefits obtained by breastfeeding	Establishment of evidence on the prevention of MTCT by STBF and FTBMF, and development of preventive measures except for feeding methods
Very low birth weight and/or very preterm infants	Banked human milk or FTBMF
Infection during pregnancy and breastfeeding after antenatal screening test	To use a contraceptive (condom)
Delivery of pregnant women who did not test antibodies during pregnancy	To test HTLV-1 antibody for these mothers as soon as possible. In the case there is an infected sibling due to MTCT, the use of infant formula may be an option to minimize the postnatal MTCT risk until the test results are obtained.

*MTCT: mother-to-child transmission, STBF: short-term breastfeeding, FTBMF: frozen-thawed breastmilk feeding.*

### Evaluation of Effect of Mother Screening on MTCT Prevention

It remains unknown whether the introduction of the screening program in Japan has contributed to a reduction in MTCT incidence at present. For this, it is necessary to examine whether children born to infected mothers become carriers. Our 2016 manual recommended to perform antibody testing in children born to carrier mothers at 3 years of age (Itabashi, 2016) because no seroconversion has been reported beyond that age (Kusuhara et al., 1987; Nyambi et al., 1996). Earlier diagnosis by serological or molecular method has been proposed (Rosadas and Taylor, 2019), but there may be little clinical advantage even if HTLV-1 infection is diagnosed.

Serological testing is not mandatory in the current screening program in Japan. A nationwide system for collecting and evaluating the results of MTCT rates in these children has not yet been established. From a public health perspective, antibody testing should be recommended for all children born to infected pregnant women. This will reveal more reliable data on the relationship between the selected feeding method and MTCT rates, and will allow us to verify the effects of introducing this screening program in Japan. On the other hand, the infected children are often asymptomatic during childhood and have difficulties predicting future HTLV-1 associated diseases at present. If future studies could predict the risk of HTLV-1-associated diseases and prevent these diseases in infected children, more children will be tested for antibodies. Healthcare providers explain the purpose of antibody testing at 3 years of age to carrier mothers using the following arguments: (1) Identification of children as carriers will allow minimization of transmission to sexual partners in the future; and (2) If you know that your child is a carrier, you will have immediate access to information when effective treatment strategies for ATL and HAM/TSP become available in the future.

### Public Awareness About HTLV-1 Infection

While a few patients have severe symptoms, most infected individuals remain asymptomatic throughout their lives and their infections may be unknown to many health providers. In addition, healthcare providers except for specialists have little experience with HTLV-1-associated diseases, and residents have little knowledge about the virus in non-endemic areas. However, as mentioned in an open letter to WHO, “HTLV-1 remains a strong threat to individual and community health, and even more so to global health because of the accelerated rate of human migration in recent times” (Martin et al., 2018). Although the nationwide antenatal HTLV-1 antibody screening program has been conducted, public awareness about HTLV-1 infection except in endemic areas still seems to be low in Japan.

### Support for Virus Carrier Mothers

Rocha-Filho and Goncalves (2018) showed both symptomatic and asymptomatic patients with HTLV-1 experienced more anxiety and depression than uninfected patients. In contrast, a study comparing HTLV between antibody positive and negative individuals do not support a biologic role for HTLV in the pathogenesis of depression and anxiety (Guiltinan et al., 2013). There is no consensus on the cause of the elevated risk of these mental disorders in HTLV-1 infected patients.

According to an interview with thirteen infected people conducted by Zihlmann et al. (2012), they stated that HTLV-1 is a largely unknown infection to society and healthcare providers due to health care providers’ inadequate responses. These investigators speculated as follows: “The diagnosis of HTLV-1 can remain a stigmatized secret as patients deny their situations. As a consequence, the disease remains invisible and there are potentially negative implications for patient self-care and the identification of infected relatives” (Zihlmann et al., 2012). It is presumed that carrier mothers may be a similar situation when they could not have sufficient support.

Little is known about the impact of the diagnosis on the mother’s emotional state (anxiety and depression), their delivery experience or the mother–infant bonding, and the relationship between the mother and her family (Rosadas and Taylor, 2019). Recent systematic review represents that breastfeeding duration is associated with postpartum depression in almost all studies. And, postpartum depression is predicted by breastfeeding cessation in several studies (Dias and Figueiredo, 2015). Therefore, it is assumed that the risk of anxiety or depression may increase in the mothers who selected ExFF or STBF not only during pregnancy but also postpartum. The Ministry of Health, Labor and Welfare has requested that prefectural governments establish a support system for carrier mothers. Carrier mothers are also concerned about their own risk for onset of ATL and HAM/TSP in the future. Carrier mothers with the risk of HTLV-1-associated diseases should be referred to a specialist physician (Ishitsuka et al., 2015).

### Elimination or Reduction of the Benefits Obtained by Breastfeeding

In developed countries, it may be possible to adopt ExFF safely for MTCT prevention because the sanitation environment is up to date. On the other hand, infants and children who have received longer term breastfeeding have lower infectious morbidity and mortality, fewer dental malocclusions, and higher intelligence than those who have been breastfed for a shorter period, or not at all. This inequality persists until later in life. Growing evidence also suggests that breastfeeding might protect against a tendency to be overweight and to develop diabetes later in life (Victora et al., 2016). A meta-analysis concluded that breastfeeding duration of at least 2 months after birth is associated with half the risk of sudden infant death syndrome. Breastfeeding does not need to be exclusive to confer this protection (Thompson et al., 2017). However, infants and children fed exclusively by infant formula may not get these benefits provided by breastfeeding.

Several risk factors for HTLV-1 MTCT other than long-term breastfeeding are known, including high mother HTLV-1 antibody titers and PVL (Ureta-Vidal et al., 1999; Hisada et al., 2002; Paiva et al., 2018). Paiva et al. (2018) reported that breastfeeding ≥12 months, higher maternal PVL (≥ 100 copies/10^4^ PBMC) and ≥ 2 previous HTLV-1-infected children were independently associated with MTCT in a multiple logistic regression. Hisada et al. (2002) suggests that mothers who have a high PVL (≥ 3%) should be encouraged not to breast-feed, while a risk of the transmission in low PVL less than 0.1% was negligible. Li et al. (2004) reported that PVL in breastmilk, which is correlates maternal PVL, is a strong predictor of risk of MTCT. However, Rosadas and Taylor (2019) mentioned that PVL in breastmilk may not be suitable because lymphocytes in breastmilk are not be main cellular population. If the infants born to only pregnant women with a high PVL would be subjected to complete formula feeding, the number of the infants fed by formula could be reduced. In order to prove this hypothesis, it would be better to conduct investigation using the antenatal HTLV-1 antibody screening program in Japan.

In the future, should it become possible to use risk factors to clearly predict the risk of MTCT, it may be possible to reduce the number of children recommended to have breastfeeding avoided or limited.

### Preventive Measures Other Than Feeding Methods

Since the 1990s, ExFF has been used as the main method to prevent postnatal MTCT. Considering the psychosocial influences carrier mothers are subjected to and the potential health risks in their infants and children associated with either completely avoiding or restricting breastfeeding, the development of additional preventive MTCT strategies such as vaccine or antiviral regimens should be developed in the future.

In animal experiments, it was reported that the administration of HTLV-1 antibody (Kuo et al., 2011; Fujii et al., 2016; Murakami et al., 2017) and the use of polyanionic microbicides are effective in preventing MTCT (Romer et al., 2009), but they are not ready for human use yet.

### Very Preterm and/or Very Low-Birth-Weight Infants Born to Carrier Mothers

The potential for viral transmission from mother to child presents a dilemma on how best to interpret the benefits and risks of breastfeeding in different settings (Prendergast et al., 2019). Meta-analysis has shown that feeding with the mother’s own milk or banked human milk can reduce the risk of necrotizing enterocolitis and/or severe infections, especially for very low-birth-weight infants (<1,500 g birth weight) or very preterm infants (<32 weeks of gestation) (Corpeleijn et al., 2016; Miller et al., 2018). Therefore, the most rational approach would be to feed banked human milk to infants born to carrier mothers for preventing not only necrotizing enterocolitis and/or severe infections but also HTLV-1 MTCT. Unfortunately, to date no human milk bank system exists in Japan. Although there is little evidence on the effect of FTBMF on the prevention of MTCT after birth, FTBMF instead of banked human milk may be the second best option because of the risk of mortality and morbidities caused by formula feeding during newborn intensive care unit admission. HTLV-1 antibodies transferred *in utero* from carrier pregnant women may offer insufficient protection in very preterm and/or very-low-birth-weight infants. We assume that FTBMF may be safer than feeding with the mother’s own milk without any treatment. However, there are few studies on MTCT in these infants to support this hypothesis.

### Pitfalls of the Nationwide Screening Program

A pregnant woman with a negative result may become infected from sexual contact with a HTLV-1-infected partner after the screening test, in which case the child could become infected by long-term breastfeeding (Nerome and Kawano, 2017). If you already know that your sexual partner is an HTLV-1 carrier, you may use a contraceptive (condom), especially during pregnancy and breastfeeding.

Not all pregnant women may have been screened for HTLV-1 antibodies during pregnancy, in which case serological antibody testing for such a woman should be performed after delivery. It is unclear whether breastfeeding during a very short period of time before the mother’s test results are obtained will increase the risk of MTCT after birth. In the case there is an infected sibling due to MTCT, the use of infant formula may be an option to minimize the postnatal MTCT risk to the newborn infant until the test results are obtained. Later, if the mother proves to be a carrier, the healthcare provider should discuss feeding methods with her.

### Follow-up of the Infected Children

Adult T-cell leukemia is generally known to be occurred in individuals with vertical infection via mainly prolonged breastfeeding, and HAM/TSP to be occurred in individuals infected via sexual intercourse or blood transfusion during adulthood. Owing to the long latency of the virus, mean onset age in ATL is 66.0 years old (Iwanaga et al., 2012). The average age of HAM/TSP diagnosed is 40 years old (Nakagawa et al., 1995).

However, several studies suggested that children infected via MTCT present with higher risk of developing ATL and/or HAM/TSP in Latin America (Murphy et al., 1989; Kendall et al., 2009; Oliveira et al., 2017). Kendall et al. (2009) showed that abnormal neurological findings (clonus and lower extremity hyperreflexia) were common in Peruvian children infected with HTLV-1. The data also suggested that persistent hyperreflexia of the lower extremities may be an early sign of HTLV-1-associated neurological involvement in children. Additionally, several cases were coprevalent with infective dermatitis. Maloney et al. (2003) reported that the childhood skin diseases associated with HTLV-1 can include seborrheic dermatitis and eczema. Oliveira et al. (2017) reviewed studies about early onset HTLV-1-associated diseases that together included 27 HAM/TSP cases and 31 ATL cases. Age at diagnosis ranged from 3 to 18 years and from 2 to 18 years for HAM/TSP and ATL cases, respectively. Interestingly, about half of HAM/TSP cases were associated with infective dermatitis. Although how the incidence of symptoms varies by age in infected children remains unknown, skin abnormalities such as seborrheic dermatitis and eczema and neurological abnormalities may appear at as early as 2 to 3 years of age. Knowing in advance that a child is a carrier would allow healthcare providers to ensure early detection of HAM/TSP and ATL. Therefore, provision of such information to the carrier mother may be helpful in encouraging antibody testing at 3 years of age or regular visits to the clinic. In addition, follow-up of MTCT pediatric carriers may help elucidate the mechanisms underlying the future development of ATL and HAM/TSP.

It remains unclear whether the association of skin lesions with HAM/TSP in HTLV-1 infected children is unique to Latin America due to a lack of studies in Japan. Yoshida et al. reported that disease onset was before 15 years of age in 10% of HAM/TSP patients in Japan (Yoshida et al., 1993). These patients shared common features of short stature and slight intellectual disability, and three of them had pseudoparathyroidism. However, no obvious signs of childhood leading to the development of HAM/TSP or ATL have been observed after their report. Therefore, little attention has been paid to symptoms in MTCT-infected children in Japan. In the future, it is desirable that antibody testing at the age of 3 is more widely performed in children born to carrier pregnant women and allow early detection of HTLV-1-associated symptoms and diseases by follow-up study.

As most infected children are asymptomatic, clinic consultation intervals and points of attention at the time of the consultation are unclear. In addition, considering the psychological effects on children, there is some debate about how old it is to be notified them to be infected. Thus, discussions are needed on how to follow up the infected children.

## Conclusion

In Japan, an antenatal HTLV-1 antibody screening program has been implemented on a nationwide scale for preventing MTCT of the virus. Pregnant women tested positive on a confirmatory or PCR test are identified as HTLV-1 carriers. Since the main infection route of HTLV-1 MTCT is breastfeeding, it is reasonable to recommend avoiding breastfeeding. Nowadays, ExFF has been considered as the most reliable method for MTCT prevention. The STBF during ≤3 months is considered if the mother is eager to breastfeed her child. However, it is important that mothers and family members fully understand not only an increase in MTCT risk with increased duration of breastfeeding but also having an insufficient evidence. As there are only a few clinical studies on the protective effect of frozen-thawed breastmilk feeding on MTCT of HTLV-1, there is little evidence to recommend this feeding method. Further study on the protective effects of STBF and FTBMF are needed.

It is assumed that the risk of anxiety or depression may increase in the mothers who selected ExFF or STBF not only during pregnancy but also postpartum. Thus, not only to provide an adequate support and counseling for these mothers in various fields but also to raise public awareness of the risks and prevention methods of HTLV-1 infection is urgently necessary. As most infected children are asymptomatic, further study is needed on how to follow up them.

## Data Availability Statement

The raw data supporting the conclusions of this article will be made available by the authors, without undue reservation, to any qualified researcher.

## Author Contributions

All authors contributed to the conception and design of the study, contributed to manuscript revisions, read and approved the submitted version. KI wrote the first draft of the manuscript.

## Conflict of Interest

The authors declare that the research was conducted in the absence of any commercial or financial relationships that could be construed as a potential conflict of interest.

## Abbreviations

ATL: adult T-cell leukemia
CLEIA: chemiluminescent enzyme immunoassay
CLIA: chemiluminescent-immunoassay electro-chemiluminescent immunoassay (ECLIA)
ExFF: exclusive formula feeding
FTBMF: frozen-thawed breast milk feeding
HAM/TSP: HTLV-1 associated myelopathy/tropical spastic paraparesis
HTLV-1: human T-cell leukemia virus type-1
LIA: Line Immunoassay
MTCT: mother-to-child transmission
PA: particle agglutination
PBMCs: peripheral blood mononuclear cells
PCR: polymerase chain reaction
PVL: proviral load
STBF: short-term breastfeeding
WB: Western Blot.
